# Defensins of *Lucilia sericata* Larvae and Their Influence on Wound Repair Processes in Practical Assessment—A Study of Three Cases

**DOI:** 10.3390/ijerph20075357

**Published:** 2023-03-31

**Authors:** Dariusz Bazaliński, Joanna Przybek-Mita, Katarzyna Lisowicz, Mateusz Skórka, Paweł Więch

**Affiliations:** 1 Father B. Markiewicz Podkarpackie Specialist Oncology Centre, Specialist Hospital in Brzozów, 36-200 Brzozów, Poland; 2 Department of Nursing and Public Health, Institute of Health Sciences, College of Medical Sciences, University of Rzeszów, 35-959 Rzeszów, Poland; 3 Department of Medical Rescue, Institute of Health Sciences, College of Medical Sciences, University of Rzeszów, 35-959 Rzeszów, Poland; 4 Postgraduate Nursing and Midwifery Education Centre, 35-083 Rzeszów, Poland; 5 Department of Nursing, Institute of Health and Economy, Carpathian State University in Krosno, 38-400 Krosno, Poland; 6 St Hedvig Clinical Provincial Hospital No. 2 in Rzeszów, 35-301 Rzeszów, Poland; 7 Department of Nursing, Institute of Health Protection, State University of Applied Sciences in Przemyśl, 37-700 Przemyśl, Poland

**Keywords:** biofilm, *Lucilia sericata*, defensins, debridement, chronic wound, MDT

## Abstract

Bacteria inhabiting chronic wounds form a biofilm that prolongs and slows down the healing process. Increasingly common antibiotic resistance requires clinicians to search for effective and alternative treatment methods. Defensins are the most common antimicrobial peptides capable of eradicating pathogens. Their discovery in maggot secretions allowed for a broader understanding of the healing mechanisms, and approving the use of *Lucilia sericata* fly larvae in the treatment of infected wounds resulted in an effective and safe procedure. The aim of the study was to present the possibility of biofilm elimination in a chronic wound by means of medical maggots (*Lucilia sericata*) with the example of three selected clinical cases. The observation included three women who met the inclusion criterion of having venous insufficiency ulcers with inhibited regeneration processes. Medical maggots were applied in a biobag for three days, and observation was conducted for 21 consecutive days. In 2 cases, a significant elimination of necrotic tissue from the wound bed with local granulation tissue was observed 72 h after application of a larvae colony on the wounds. In 1 case, the application of the larvae accelerated the repair process by reducing the wound area by approximately 40% at the time of observation. The formation of biofilm in a chronic wound is one of the main causes of disturbances in its effective healing. Combining procedures (scraping, antiseptic compresses, MDT, NPWT) related to wound debridement increases the effectiveness of biofilm elimination. The use of medical maggots is a safe and effective method of choice, and it enhances the processes of debridement. However, confirmed indisputable data on their effectiveness and frequency of use in the process of stimulating healing processes are still not available in the literature.

## 1. Introduction

Damage to the skin and subcutaneous tissue is a common problem associated with injuries as well as chronic systemic processes causing disturbance in blood supply and oxygenation of tissues. This problem is more and more common and affects nearly 2% of the population of developed countries, which is a serious socio-economic issue [1]. The healing of uncomplicated wounds is subject to complex and dynamic processes involving successive phases related to hemostasis, inflammation, proliferation, and tissue remodelling [2]. The repair phases usually proceed in a predictable manner, resulting in tissue healing without the need for significant intervention. Wounds that do not respond to treatment in accordance with accepted standards and remain in a prolonged inflammatory-proliferative phase are defined as difficult to heal [3]. Prolonged healing processes lasting more than 6 weeks give the basis to label the wound as chronic (the exception of 14 days or more is a wound in the course of a diagnosed diabetic foot) [4,5]. The wound hygiene concept developed by European experts was based on the assumption that biofilm-forming microbes are the main cause of delayed healing in 60–90% of cases, which is noticeable just a few days after the potential injury [3,6,7,8]. Biofilm is defined as a highly structured, three-dimensional cluster of microorganisms (bacteria or fungi) embedded in a self-produced extracellular polymeric substance (EPS) [9,10,11,12]. The formation of a biofilm system is a multi-stage process, depending on the structure and physicochemical properties of the colonized surface. Detachment of bacterial cells from the formed structure and their circulation with blood or other body fluids is both the last stage of biofilm development and the beginning of the expansion of new surfaces [6]. The biofilm matrix surrounding the bacteria makes them tolerant to harsh conditions and resistant to antimicrobial treatment. The emergence of antibiotic resistance in them reduces the effectiveness of the treatment. The cells that make up the biofilm have different properties than those existing in free form. Planktonic phenotype infections are more aggressive and violent. Nevertheless, the metabolic activity of pathological cells is higher and the risk of antibiotic resistance is lower. Its formation affects both biotic and abiotic surfaces; it may also not adhere to any surface [3,8,13,14]. The available antibiotics may be ineffective in treating these infections due to their higher minimum inhibitory concentration (MIC) and minimum bactericidal concentration (MBC) values, which can cause in vivo toxicity [1]. The basic local action resulting from the wound hygiene concept is systematic debridement, associated with the elimination of devitalized tissues from the wound surface. This process can be carried out by means of various methods and techniques as well as substances with an antiseptic effect recommended by scientific societies [15,16]. Over the last decade *Lucilia sericata* medical larvae have been claimed to be “miracle therapeutic maggots” due to their manifold biochemical properties that stimulate healing processes in a wound. Isolating chemical substances from maggot excretions and secretions gives greater possibilities to develop research on the use of defensins to stimulate healing processes in wounds of different aetiologies. Several randomized trials and meta-analyses confirm high effectiveness of secretions and excretions produced by larvae in the process of wound debridement and healing [17,18]. Excretions and secretions (ES) of the larvae contribute to the elimination of bacteria and stimulate repair processes. The anti-biofilm and antibacterial effect of protein substances excreted by the larvae is visible during local therapy. Tissue renewal and reconstruction is clearly seen. The authors indicate that (ES) strongly inactivates *Pseudomonas aeruginosa*, methicillin-resistant *Staphylococcus aureus* (MRSA), and *Streptococcus A and B* [18,19,20].

The aim of the study was to present the use of *Lucilia sericata* medical maggots in chronic wounds located in the area of the lower legs on the example of three selected clinical cases.

## 2. Materials and Methods

Out of a group of 30 patients with lower leg ulcers in the course of chronic venous insufficiency (CVI) treated in the wound care clinic in 2021, 3 female patients, aged 64, 68, and 87 years (mean age 73 years), were randomly selected by means of Excel pseudo-random number generator. The selected subjects demonstrated features of regression in the healing process (no epithelialization; yellow fibrinous devitalized tissue in the wound; they were not qualified for surgical treatment with tissue graft; their nutritional status was normal; blood biochemical parameters (hemoglobin, albumin, creatinine, glucose) and markers of systemic infection (CRP, PLT) were within normal range; the ankle brachial index (ABI) was over 0.8; compression therapy was implemented). In the microbiological evaluation, *Staphylococcus aureus* (+++) (Case I and II) and *Pseudomonas Aeruginosa* (+++) (Case III) were found. The time since the occurrence of the wounds ranged from 2 to 10 years. The treatment in the clinic did not exceed 12 months (2–12 months). Microbiological evaluations were collected from wounds 7–10 days before the planned implementation of MDT. Re-inspection was performed after 30 days. In every case, the recommended debridement methods were used (scraping, active dressings, antiseptic gels). The patients were informed orally and in writing about the scope of potential complications and therapeutic measures for which they consented based on the Helsinki declaration (Bioethics Committee 2017). The level of biodebridement acceptance was assessed using the MDT acceptance questionnaire [21]; each of the respondents presented low values in the questionnaire assessment, and therefore a closed form of larvae in the biobag was implemented. They also had the opportunity to directly contact the person responsible for monitoring the therapeutic process by phone. Maggot debridement therapy (MDT) in the biobag 10 × 10 (approx. 120 pcs of larvae) (Biofenicja) Biomantis, Krakow, Poland, was implemented. The application and supervision were in line with the guidelines of PTLR (Polish Wound Treatment Society) 2020 [22]. During the three-day therapy, the dressing was moistened with sterile 0.9% NaCl solution once a day; no compression therapy was used during this period. A visual assessment of the wound condition was performed before and after debridement assessing the ratio of viable (red) granulation tissue to (yellow) devitalized/necrotic tissue. Based on the percentage of wound contamination with necrosis, the “debridement index” was calculated in all patients before and after debridement by means of the Equation:

$$\mathrm{debridement}\;\mathrm{index}=100-\frac{x_{1}}{x_{2}}\times 100$$

where:

x_1_—the percentage of necrosis and purulent exudate before treatment.

x_2_—the percentage of necrosis and purulent exudate after treatment.

The obtained results were classified into separate percentage ranges, where:

0—no debridement of necrotic tissues (lack of therapeutic effect).

10–30%—poor wound debridement (unsatisfactory therapeutic effect).

40–80%—average debridement (good therapeutic effect).

90–100%—complete debridement of the wound (very good therapeutic effect) [21].

## 3. Results

### Description of the Cases

**Figure 1:**
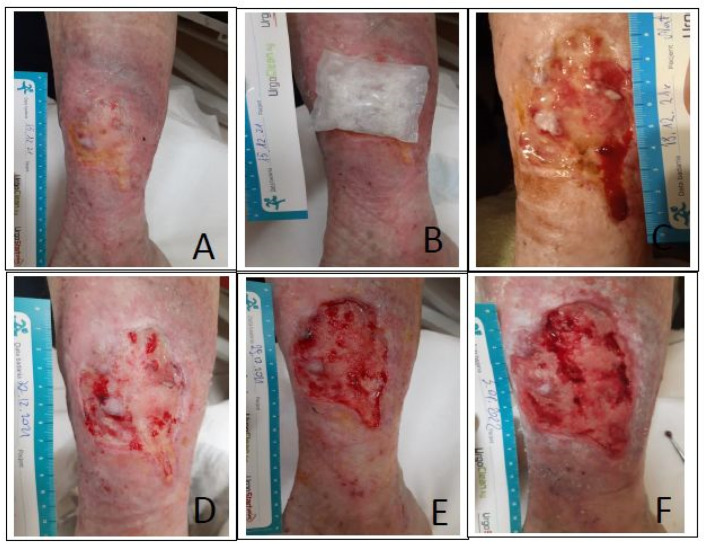
The course of the treatment process with medical maggot biobag in case no. 1. (**A**) The wound before the application of the larvae, with visible healing inhibition process fibrin in the wound hard dense without the possibility of evacuation using scraping. (**B**) Application of the larvae in the biobag—100 pcs. (**C**) Condition of the wound after 7 days: slight granulation process, scarce exudation, no pain, change of dressings (polyurethane plus antiseptic gel every 3 days plus scraping). (**D**), (**E**) Condition of the wound after 14 days after scraping: the significant process of fibrin formation and deposition in the wound, low exudate. The concept of local treatment was changed to hydroactive dressings plus antiseptic gel. (**F**) Condition of the wound after 21 days: visible “lazy” granulation tissue in the wound and scarce epithelialization and healing process unsatisfactory.

Case I:

Case I was a woman aged 64 capable of self-care (score 80 in Barthel Index) with a history of CVI in the left lower leg wound for over 10 years. For 10 months she was treated at the wound treatment clinic, and the wound was reduced by 50%. For several weeks, inhibition of granulation processes, reduction of exudate, and hard yellow devitalized tissue were present in the wound. At the time of qualification for the study, the lesion area was over 50 cm^2^, of full-skin thickness damage, classified as a yellow wound (according to RYB), with scarce exudate (in the microbiological assessment *Staphylococcus aureus* (++) strain MSSA (methicillin-sensitive Staphylococcus aureus) was found), and without reported pain. Additionally, compression therapy II° and auxiliary mesotherapy (once a week with a collagen-based preparation) of pressure were implemented. A biobag (100 larvae) was used for a period of 3 days, and each day the dressing was inspected. During the therapy, the patient did not report any pain above 3 points (NRS); the wound debridement was 50% (good therapeutic effect). Then, foam dressings plus an antiseptic gel were implemented along with the continuation of compression therapy with a change of sequence every 3 days and scraping the wound once a week during the follow-up at the clinic. After 14 days of MDT, hydroactive dressings were introduced due to the symptoms of the so-called “dry wound” (Figure 1), the patient was offered to consider NPWT (negative pressure wound therapy). Poor healing and reduction of the wound area was observed within 21 days of MDT.

**Figure 2:**
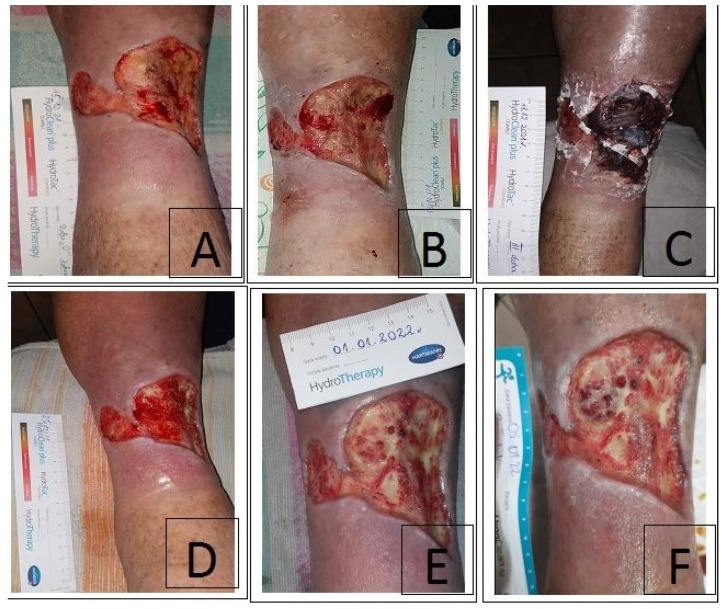
The course of the treatment process with *Lucilia sericata* larvae in the biobag in case no. 2. (**A**) Wound after scraping before larvae application: visible healing inhibition, locally dense hard fibrin without the possibility of thorough debridement using mechanical and surgical methods. (**B**) Second day of MDT in the biobag—3 bags with 100 pcs. (**C**) Third day of the MDT—visible signs of capillary bleeding, which prompted the completion of the wound debridement process with this method. (**D**) State of the wound after 7 days: slight granulation process, moderate exudate, moderate amount of fibrin, easy for scraping, pain only during debridement, change of dressings (TLC-NOSF polyacrylate + antiseptic gel every 3 days plus scraping). (**E**) Condition of the wound after 14 days: weak granulation process visible, scarce exudate. (**F**) Condition of the wound after 21 days: no pain, more and more clumps of granulation tissue, healing process unsatisfactory.

Case II:

Case II was an 87-year-old woman capable of self-care (score 90 in Barthel Index) with a history of CVI, cardiostimulator, and novel oral anticoagulants (NOAC), with an ulcerated wound in the right lower leg for about 4 years. The patient was treated at the wound treatment clinic for about 3 months. On examination, a full-thickness wound of the skin over 50 cm^2^ was found which was red–yellow (according to RYB), with a Wound at Risk (WAR) score 3, no features of healing, scarce exudate (in the microbiological assessment *Staphylococcus aureus* (++) was found). Additionally, II° compression therapy was initiated, and the wound was mechanically debrided and then silver foam dressings plus antiseptic gels were introduced. A biobag (3 × 100 larvae) was used on the prepared medium for a period of 3 days and monitored every 24 h. The patient did not report any significant pain symptoms: 2–3 points (NRS/VAS) during the therapy on day “0”, and an increase in the level of pain was observed in the following days to 7–8 points (NRS/VAS). On the third day of therapy, bleeding was noted from the wound, the wound was revised, the biobags were evacuated, and a hemostatic dressing was applied. Wound debridement was within 70% (good therapeutic effect). Then, alginate plus foam dressings and an antiseptic gel were implemented along with the continuation of II° compression therapy and the sequence of dressing changes every 3 days; wound scraping was performed once a week during the inspection (Figure 2). Slow progression of healing and granulation growth within the wound was noted during 21 days of MDT. Next, the patient was prepared for NPWT therapy.

**Figure 3:**
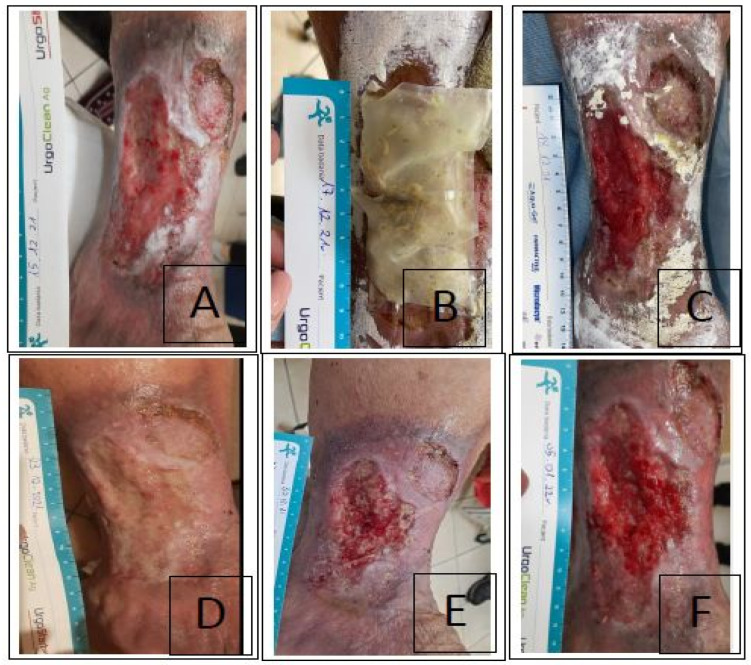
The course of the treatment process with the use of *Lucilia sericata* larvae in the biobag in case No. 3. (**A**) Wound after scraping before larvae application: visible healing inhibition, local hard compacted fibrin, edema and discoloration of the limb. (**B**) Second day of MDT in the biobag—2 bags with 100 pcs. (**C**) Third day of MDT—wound practically 100% debrided. (**D**) Condition of the wound after 7 days: (without scraping) slight granulation process, moderate exudate, moderate amount of fibrin, easy for scraping, pain only during debridement, foam dressings plus antiseptic gel. (**E**) Condition of the wound after 14 days: visible granulation process, low exudate. (**F**) Condition of the wound after 21 days: no pain, visible granulation and skin formation, reduction of the wound area, satisfactory healing process.

Case III:

Case III was a 68-year-old woman capable of self-care (score 80 in Barthel Index) with a history of CVI in the left lower leg wound for over 2 years (not referred to compression therapy). The patient treated at the wound treatment clinic for about 2 months. Swollen limbs, full-thickness wound of the skin over 50 cm^2^ which was red–yellow (according to RYB), WAR above 3 points, lack of healing features, medium exudate in the microbiological assessment of *Pseudomonas Aeruginosa* (+++). Additionally, II compression therapy was initiated, mechanical debridement was performed, and then antiseptic dressings based on povidone iodine were implemented (PVP-I), plus foam dressings. A biobag (2 × 100 larvae) was used for 3 days and the dressing was inspected each day. Then, foam dressings plus an antiseptic gel were implemented along with the continuation of II° compression therapy with a change in sequence every 3 days and scraping the wound once a week during the follow-up at the clinic (Figure 3). A visible healing process and reduction of the wound area were observed within 21 days after MDT application.

Detailed data on wounds before MDT and o follow-up after 21 days after MDT implementation are presented in Table 1 and Table 2.

## 4. Discussion

One of the major issues concerning the elimination of biofilm is increasing antibiotic resistance. A meta-analysis by Malone et al. confirms the presence of biofilm in 78.2% of chronic wounds [24]. Too frequent and unjustified antibiotic therapy contributes to the development of persisting cells—a subpopulation of surviving cells thus enabling the reconstruction of the biofilm population [7,25]. Less than 4 years (1944) after the introduction of penicillin to the pharmaceutical market, a β-lactamase-producing strain was noted in over 50% of samples with *Staphylococcus aureus*, which clearly proved the development of resistance to most of the available antibiotics. Growing antibiotic resistance is accompanied by a negative aspect in terms of time and economy—the therapy becomes longer, additionally increasing the overall costs of patient care and the overall financial expenses of the society [26]. Multi-drug resistant organisms (MDRO) have become a serious threat to civilization, stimulating the search for more effective methods of destroying microorganisms. Insightful observations and research by Sherman and Pechter on the elimination of bacterial flora, including MRSA (methicillin-resistant *Staphylococcus aureus*), by larvae placed in the wound opened up new opportunities for researchers and clinicians all over the world [27].

According to the guidelines, the elimination of devitalized necrotic tissue is the basic procedure in the treatment and management of a wound [3,17,26,27]. Debridement of a wound in which repair processes are inhibited is not subject to unambiguous “rigid” guidelines. The method of non-physiological tissue elimination is multifactorial and related to the area, location and depth of damaged structures, the amount of exudate, concomitant pain, as well as the general condition of the patient and their preferences [16,28]. Mechanical debridement of the wound (rubbing, scraping, plucking, cutting out) is the simplest, cheapest and fastest method of biofilm elimination performed by trained medical personnel [3,8,9,10,17]. However, in most chronic wounds with coexisting biofilm, more advanced preventive measures are required, and intervention in the form of autolytic biological interventions should be considered, and then the implementation of controlled negative pressure wound therapy in local wound therapy needs to be implemented [27,29]. Taking into account expert recommendations and clinical observations, three wounds with an area of approx. 50 cm^2^ colonized with microorganisms were subjected to biological debridement from devitalized tissues and monitoring of subsequent repair processes. The subjects were randomly selected from a sample of 30 people who had MDT used in the course of topical wound treatment based on specific criteria. Larvae in a biobag were used due to less pain and aversion of patients related to the sight of worms. It was taken into account that the wound debridement options may be less effective, but they reduce the psychological fears of the subjects. The expected quick debridement of the wound was noted, however, a few days after debridement, two out of three wounds (footnotes 1 and 2) were still not healing properly, covering themselves with fibrin. In line with the recommendations, scraping was used, antiseptic was applied before changing the dressing, compressions were performed, and foam dressings soaked with antiseptic. The above observations, which were recorded in the presented patients as well as in the remaining group of respondents, indicate that the implementation of MDT should be a standard and repeated in 7–10 day cycles in order to minimize bacteria and biofilm as well as to stimulate repair processes in the wound. The above clinical observations are consistent with the results of research conducted by Akbas et al., who proved that larval defensins containing fumaric acid, ferulic acid, and p-coumaric acid enhanced migration of fibroblasts and can modulate mRNA expression of some genes related to the wound healing process [30].

In a meta-analysis by Sun et al., MDT not only shortened the healing time but also improved the healing rate of chronic ulcers [31]. The biological debridement of the wound is related to the mechanical and biochemical effects associated with the protein defensins produced by maggots. Mechanical debridement is associated with the removal of necrotic tissue and maggots wriggling in the wound area (which may arise sense of tingling and even pain). Getting rid of necrosis from the wound increases oxygen availability to healthy tissues, facilitates the migration of fibroblasts and keratinocytes, and physically eliminates pathological microorganisms, which reduces the likelihood of their further multiplication [19,28,31,32,33]. Maggot wriggling in the wound bed stimulates neoangiogenesis and granulation. Restoring the functional vascular network is fundamental in diabetes foot syndrome. The study by Sun et al. indicates a number of neoangiogenic factors, including those activating endothelial cells [33]. Analysis of wounds before and after the application of medical maggots proves the promotion of wound healing on many levels, and hence the interesting element of MDT is a broad chemical action based on the secretion and excretion of specific enzymes and their correlation with antibacterial activity (Lucilin, Lucifensin, Lucifensin II, MAMP (Alpha -methoxyphenol), (seraticin) [34,35,36,37,38,39] antibiofilm (Chymotrypsin) [40,41,42], anti-inflammatory; excretions/secretions—ES) [40], synergism with selected antibiotics, and immunomodulatory functions [41,42]. Human skin also has the ability to produce defensins. Antimicrobial peptides (AMPs) are one of the primary mechanisms used by the skin in the early stages of immune defence. AMPs have a broad antibacterial as well as anti-fungal and antiviral effects. In their study, Fijałkowska et al. showed the relationship between the presence of basal cell carcinoma (BCC) cells and the concentration of cathelicidin and β-defensins in plasma (HBD1-3). Elevated levels of cathelicidin and β-defensin 2 are associated with the presence of BCC. The specificity of cathelicidin and β-defensin 2 in the detection of BCC was confirmed, which in the future may be a determinant in the assessment of risk cancer. The authors indicate that these factors are not specific only to this disease and further studies are required [43].

The antimicrobial activity of maggots was also observed in the case of bacteria characterized by high resistance to antibiotics, such as *Pseudomonas aeruginosa* and *Staphylococcus aureus* [37,38,39,44]. The elimination of biofilm in these cases is particularly important due to the high resistance to penetration and the action of the human immune system and to antibiotics [45,46]. An important discovery in recent years is the fact that lucifensin with antibacterial properties is not found in the digestive tract of *Lucilia sericata* but in the salivary glands and the fat body. It has also been proven that the larvae generate an immune response to the infectious environment in which they reside by increasing the expression of lucifensin in the fat body, from which it is secreted into the hemolymph. Alatonin excreted by maggots promotes local and temporary proliferation of leukocytes. The presence of it and other substances such as ammonium bicarbonate and urea are believed to keep the wound pH in the alkaline range necessary for the activity of *Lucilia sericata* proteases during debridement. As the wound heals, the pH shifts from alkaline to neutral until it is acidic in healthy skin. Szczepanowski et al. drew attention to the fact that debridement of wounds with larvae also changes the bacterial flora in the wound; the authors indicate that *Proteus mirabilis* is a microorganism present in the maggot’s digestive tract and may contaminate the wound [47]. In our material, a microbiological assessment of wounds was performed after a period of not less than 30 days, confirming the eradication of *S. aureus* but with a scanty growth of *Proteus mirabilis*. It was not possible to eradicate *Pseudomonas aeruginosa* in the observed time; however, the wound demonstrated healing processes and was finally healed, and the remaining wounds could not be healed, but their area and depth decreased by more than 50% in the 12-month follow up.

In the analyzed cases, wound scrapings were collected for microbiological testing before the implementation of MDT, and no scrapings were collected until 7 days after debridement, which we consider to be a limitation of this study. Observations related to periodic sterilization of the wound or contamination of *Proteus mirabilis* are our frequent observations as well as those reported by other authors [20,47,48]. Microbiological material was collected only 20 days after MDT application and still indicated bacterial colonization. Nezakati et al. reported that larvae therapy has a varied effect on bacterial species, eliminating *P. aeruginosa*, *E. coli,* and *S. aureus* and having the least impact on the growth of *Enterococcus*, thus stressing that research should be extended in this direction [48].

**Figure 4:**
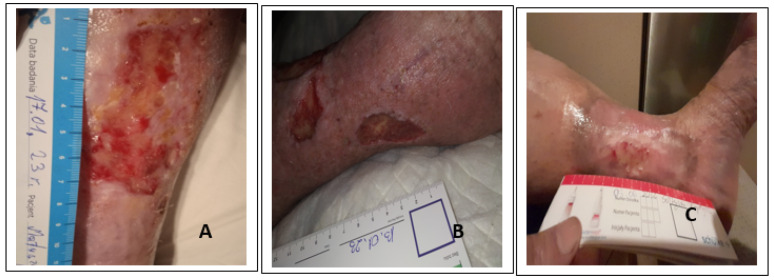
The photographs demonstrate the condition of the wounds in follow up after a minimum of 10 months, with patients still under the care of the wound care clinic. Case 3 (**C**) practically healed without pain. Case 2 (**B**) wound with partial skin thickness healed over 50%, with age-related limitation of self-care and circulatory failure. Case 1 (**A**) partial-thickness wound without signs of infection reduction by 50%, active, no pain, non-professional care for the sick husband.

Complications related to the use of biological debridement are rare and mainly relate to intense mental and somatic sensations (irritation of nerve endings) generating pain and anxiety. In the discussed cases, the subjects tolerated the therapy well on the first day, but on the second and third days they reported the intensification of pain above 4 points, and therefore non-opioid analgesics were prescribed in a protocol. Bleeding was observed in one of the subjects taking NOAC, which was related to the opening of a small vessel and capillary bleeding, which worried the patient. Patients using anticoagulants should not be disqualified from this form of wound debridement; particular attention should be paid to the observations reported by the patient during the therapy. To sum up, in only one of the examined cases, the acceleration of the repair processes and the reduction of the wound area by about 40% were confirmed. In the remaining cases, the appearance of granulation tissue was noted but with no signs of visual epithelialization or reduction of the wound area. Nevertheless, the above observations clearly indicate a positive effect of the action of medical maggots in regenerative processes. Carrying out the overall analysis, the conclusion is that this form of debridement should be used more often (every 7 days) when the wound begins to be covered with devitalized fibrin tissue, which suggests the formation of a bacterial biofilm. Analyzing the cases of patients, an unequivocal conclusion arises that patients after wound preparation (cases I and II) (granulated tissue with minimal bacterial growth) should be re-consulted regarding the possibility of covering with autogeneous tissue, with broadly understood education and indicating the advantages of this method and potential disadvantages. Repair processes in the skin tissue in elderly people with coexisting chronic diseases may be disturbed. Molecular mechanisms associated with chronic venous disease and venous hypertension lead to severe lipodematosclerotic, structural, and functional changes in the lower leg, leading to inflammation, interruption of keratinocyte migration, and abnormal regulation, signalling, and/or expression of specific micro RNAs. In order for local treatment to be effective, the functionality of tissues and circulation in the limbs should be improved (elimination, minimization of microorganisms, reduction of edema, improvement of nutrition) [49]. Skin grafting is one of the most common surgical procedures performed to shorten the healing of a chronic wound, consisting in covering the debrided wound bed with autologous tissue taken from another area of the body. This form of treatment can be implemented in the group of patients with efficient peripheral circulation, not treated with glucocorticoids or cytotoxic drugs. All patients undergoing skin grafting should be educated on the risk of graft failure. Chronic leg ulcers are a significant problem entailing major costs in Western countries and requiring various management strategies [50]. Skin grafting is a treatment method that can reduce the area of chronic leg ulcers or heal them completely in a short period of time, thus improving the patient’s quality of life. Currently, skin grafts play a key role in the context of modern wound healing and tissue regeneration. Although autologous split-thickness skin grafts (STSG) still remain the gold standard in terms of safety and efficacy in the treatment of chronic leg ulcers, in practice, the possibilities may be limited by the patient, i.e., their fear of failure, formation of a larger wound area, reluctance to hospitalize, limited access to professional care, inadequate level related to the method [51]. In our study, wounds were subjected to debridement; however, two patients did not agree to use the method. The oldest patient was not eligible due to age and limited changes in arteries. As a result, the process of local treatment was significantly extended, and the control after a few 12 month follow-ups showed the practical healing of one wound and the reduction of the area of two wounds by more than 50% (Figure 4).

According to specialists, MDT is beneficial and perspective in the treatment of wounds. The presented cases were randomly selected and we did not achieve complete success with the use of MDT; however, our observations are consistent with Sherman’s reports [52,53]. To achieve better results, the application should be repeated more often, which will allow us to reduce the biofilm and improve repair processes in the wound at relatively lower costs and greater patient acceptance of the method. In parallel, as new biomechanisms are discovered, statistical analyses of the speed of debridement and healing of wounds are being carried out. The use of larvae can also reduce the overall cost of treating difficult-to-heal wounds, reducing or excluding hospital stay and antibiotic consumption. In our opinion, medical maggots can be used for debridement and revitalization of tissues before further surgical management related to the surgical treatment of ulcers. During the COVID-19 pandemic, this form of wound debridement becomes not only an alternative but also a necessity for patients who, for various reasons, cannot have a surgically debrided wound in hospital conditions [28,31,54].

## 5. Conclusions

The wound healing process is complex and multifactorial. All possible recommended local treatments that reduce the bacterial count and create conditions for healing by granulating or covering the wound with tissue material should be considered. Formation of a bacterial biofilm in a chronic wound is one of the main causes of disturbances in its effective healing. Combining procedures (scraping with subsequent antiseptic application, MDT, NPWT) related to wound debridement increases the effectiveness of bacterial biofilm elimination. The use of medical maggots is a safe and effective method of choice, and it enhances the processes of debridement. However, there is still a lack of confirmed, indisputable data on the effectiveness and frequency of use in the process of stimulating healing processes.

## 6. Limitations

The presented cases were randomly selected, and the healing process was presented during a 21-day follow up. Given that these were infected wounds, it is too short a time to draw constructive conclusions. Although there were no spectacular effects, the impact of the MDT method was assessed positively in each case. The patients presented in the study were not qualified for the surgical management of tissues with split-thickness skin graft (STSG) due to the lack of consent to this form of treatment.

## Figures and Tables

**Table 1:** Data on wound characteristics before MDT implementation.

	Wound Surface (cm^2^)	Tissue Damage	RYB	Infection acc. to NERDS/STONES [23]	CRP (0–5 mg/L)	Exudation	ABI R/L
Case I Woman, age 64	55	full thickness of the skin	Yellow	YES/NO	25	medium	(0.9)/(1.0)
Case II Woman, age 87	78	full thickness of the skin	Yellow	YES/NO	76	large	(0.8)/(0.8)
Case III Woman, age 68	86	full thickness of the skin	Yellow	YES/NO	84	large	(0.8)/(0.9)

NERDS—features of superficial infection (lack or poor healing, exudate, exuberant granulation tissue, necrotic tissue). STONES—features of deep infection (wound enlargement, increase in temperature in the wound, exposed tendon, bone, stench, increased exudate, phlegmon, new area of damage) [23].

**Table 2:** Data on wound characteristics on follow-up after 21 days after MDT implementation.

	Wound Surface (cm^2^)	Tissue Damage	RYB	Infekcja wg NERDS/STONES	CRP (0–5 mg/L)	Exudation	ABI R/L
Case I Woman, age 64	50	full thickness of the skin	Red	NO/NO	15	small	(0.9)/(1.0)
Case II Woman, age 87	78	full thickness of the skin	Red/Yellow	YES/NO	56	medium	(0.8)/(0.8)
Case III Woman, age 68	62	full thickness of the skin	Red	NO/NO	24	small	(0.8)/(0.9)

## Data Availability

The data presented in this study are available on reasonable request from the corresponding author: pwiech@ur.edu.pl.

## References

[1] Le Goff-Pronost M., Bénédicte M., Jean-Pierre B., Teot L., Hervé Benateau H., Dompmartin A. (2018). Real-World Clinical Evaluation and Costs of Telemedicine for Chronic Wound Management. Int. J. Technol. Assess. Health Care.

[2] Gurtner G.C., Werner S., Barrandon Y., Longaker M.T. (2008). Wound Repair and Regeneration. Nature.

[3] Atkin L., Bućko Z., Conde Montero E., Cutting K., Moffatt C., Probst A., Romanelli M., Schultz G.S., Tettelbach W. (2019). Implementing TIMERS: The race against hard-to-heal wounds. J. Wound Care.

[4] Mospan B., Junka A., Bartoszewicz M. (2018). Nowe oblicze znanych związków w postępowaniu miejscowym w ranach przewlekłych. [A new character of known compounds in topical treatment of chronic wounds]. Leczenie Ran.

[5] Bartoszewicz M., Krasowski G., Banasiewicz T., Lipiński P., Bielecki K., Chrapusta A., Korzon-Burakowska A., Kucharzewski M., Mospan B., Konrady Z. (2020). Wskaźnik terapeutyczny miejscowego zakażenia rany (TILI) jako przydatne narzędzie w efektywnej pielęgnacji ran niegojących się dla lekarzy i pielęgniarek podstawowej opieki zdrowotnej, lekarzy rodzinnych i personelu zakładów opiekuńczo–leczniczych. [The therapeutic index of local wound infection (TILI) as a useful tool in the effective care of non-healing wounds for doctors and nurses of primary care, family doctors and staff of care and treatment facilities]. Forum Zakażeń.

[6] Czyżewska-Dors E., Dors A., Pomorska-Mól M. (2018). Właściwości biofilmu bakteryjnego warunkujące oporność na antybiotyki oraz metody jego zwalczania. [Properties of bacterial biofilm determining resistance to antibiotics and methods of combating it]. Życie Weterynaryjne.

[7] Maciejewska M., Bauer M., Dawgul M. (2016). Nowoczesne metody zwalczania biofilmu bakteryjnego. [Modern methods of combating bacterial biofilm]. Post. Mikrobiol..

[8] Thaarup I.C., Iversen A.K.S., Lichtenberg M., Bjarnsholt T., Jakobsen T.H. (2022). Biofilm Survival Strategies in Chronic Wounds. Microorganisms..

[9] Li Y.-H., Tian X. (2012). Quorum sensing and bacterial social interactions in biofilms. Sensors.

[10] Wu H., Moser C., Wang H.-Z., Høiby N., Song Z.-J. (2015). Strategies for combating bacterial biofilm infections. Int. J. Oral. Sci..

[11] Kadam S., Shai S., Shahane A., Kaushik K.S. (2019). Recent Advances in Non-Conventional Antimicrobial Approaches for Chronic Wound Biofilms: Have We Found the ’Chink in the Armor’?. Biomedicines.

[12] Bartoszewicz M., Banasiewicz T., Bielecki K. (2019). Zasady postępowania miejscowego i ogólnego w ranach/owrzodzeniach objętych procesem infekcji. [Principles of local and general management in wounds/ulcers covered by the infection process]. Forum Zakażeń.

[13] Karolewska K., Wójcik U., Sadowska B., Różalska B. (2018). Przegląd nowoczesnych technik obrazowych i analitycznych w badaniu cech biofilmów. [Review of modern imaging and analytical techniques in the study of biofilm features]. Forum Zakażeń.

[14] Franklin M.J., Chang C., Akiyama T., Bothner B. (2015). New Technologies for Studying Biofilms. Microbiol Spectr..

[15] Mrozikiewicz-Rakowska B., Mieczkowski M., Głażewski T., Czupryniak L. (2020). Antyseptyki w leczeniu ran przewlekłych-Aktualne pytania. [Antiseptics in the treatment of chronic wounds-Current questions]. Leczenie Ran.

[16] Kramer A., Dissemond J., Kim S., Willy C., Mayer D., Papke R., Assadian O. (2018). Consensus on Wound antisepsis: Update 2018. Ski. Pharm. Physiol..

[17] Mohd Zubir M.Z., Holloway S., Mohd Noor N. (2020). Maggot Therapy in Wound Healing: A Systematic Review. Int. J. Environ. Res. Public Health.

[18] Sherman R.A. (2014). Mechanisms of maggot-induced wound healing: What do we know, and where do we go from here? Evid. Based. Complement. Alternat. Med..

[19] Yan L., Chu J., Li M., Wang X., Zong J., Zhang X., Song M., Wang S. (2018). Pharmacological Properties of the Medical Maggot: A Novel Therapy Overview. Evid Based Complement Altern. Med..

[20] Opletalová K., Blaizot X., Mourgeon B., Chêne Y., Creveuil C., Combemale P., Laplaud A.-L., Sohyer-Lebreuilly I., Dompmartin A. (2012). Maggot therapy for wound debridement: A randomized multicenter trial. Arch. Derm..

[21] Bazaliński (2019). Skuteczność terapii biologicznej z wykorzystaniem larw Lucilia sericata w leczeniu ran przewlekłych u chorych w opiece długoterminowej i paliatywnej. Efficacy of Biological Therapy with Lucilia Sericata Larvae in the Treatment of Chronic Wounds in Patients in Long-Term and Palliative Care.

[22] Szewczyk M.T., Cwajda-Białasik J., Mościcka P., Cierzniakowska K., Bazaliński D., Jawień A., Spannbauer A., Polak A., Sopata M., Kozłowska E. (2020). Treatment of pressure ulcers—Recommendations of the Polish Wound Management Association. Part II. Leczenie Ran..

[23] Sibbald R.G., Woo K., Ayello E. (2007). Increased bacterial burden and infection: NERDS and STONES. Wounds.

[24] Malone M., Bjarnsholt T., McBain A.J., James G.A., Stoodley P., Leaper D., Tachi M., Schultz G., Swanson T., Wolcott R.D. (2017). The prevalence of biofilms in chronic wounds: A systematic review and meta-analysis of published data. J. Wound Care.

[25] Ciofu O., Rojo-Molinero E., Macià M.D., Oliver A. (2017). Antibiotic treatment of biofilm infections. APMIS.

[26] Gupta S., Andersen C., Black J., Fife C., Lantis J.I., Niezgoda J., Snyder R., Sumpio B., Tettelbach W., Treadwell T. (2017). Management of chronic wounds: Diagnosis, preparation, treatment, and follow-up. Wounds.

[27] Sherman R.A., Pechter E.A. (1988). Maggot therapy: A review of the therapeutic applications of fly larvae in human medicine, especially for treating osteomyelitis. Med. Vet. Entomol..

[28] Bazaliński D., Kózka M., Karnas M., Więch P. (2019). Effectiveness of Chronic Wound Debridement with the Use of Larvae of Lucilia Sericata. J. Clin. Med..

[29] Hurlow J.J., Humphreys G.J., Bowling F.L., McBain A.J. (2018). Diabetic foot infection: A critical complication. Int. Wound J..

[30] Akbas F., Ozaydin A., Polat E., Onaran I. (2020). *Lucilia sericata* Larval Secretions Stimulating Wound Healing Effects on Rat Dermal Fibroblast Cells. Rec. Nat. Prod..

[31] Sun X., Jiang K., Chen J., Wu L., Lu H., Wang A., Wang J. (2014). A systematic review of maggot debridement therapy for chronically infected wounds and ulcers. Int J Infect Dis..

[32] Szczepanowski Z., Tukiendorf A., Krasowski G. (2021). Further Data on Wound Healing Rates After Application of Lucilia sericata. Int. J. Low. Extrem. Wounds..

[33] Sun X., Chen J., Zhang J., Wang W., Sun J., Wang A. (2016). Maggot debridement Therapy promotes diabetic foot. Wound healing by up-regulating endothelial cell activity. J. Diabetes Its Complicat..

[34] Van der Plas M.J., Jukema G.N., Wai S.W., Dogterom-Ballering H.C., Lagendijk E.L., Van Gulpen C., Nibbering P.H. (2008). Maggot excretions/secretions are differentially effective against biofilms of *Staphylococus aureus* and *Pseudomonas aeruginosa*. J. Antimicrob. Chemother..

[35] Margolin L., Gialanella P. (2010). Assessment of the antimicrobial properties of maggots. Int. Wound J..

[36] Bexfield A., Bond A.E., Roberts E.C., Dudley E., Nigam Y., Thomas S., Ratcliffe N.A. (2008). The antibacterial activity against MRSA strains and other bacteria of a <500Da fraction from maggot excretions/secretions of Luciliasericata (Diptera: Calliphoridae). Microbes Infect..

[37] Zhang Z., Wang J., Zhang B., Liu H., Song W., He J., Lv D., Wang S., Xu X. (2013). Activity of antibacterial protein from maggots against staphylococcus aureus in vitro and in vivo. Int. J. Mol. Med..

[38] Pritchard D.I., Brown A.P. (2015). Degradation of MSCRAMM target macromolecules in VLU slough by Lucilia sericata chymotrypsin 1 (ISP) persists in the presence of tissue gelatin as activity. Int. Wound J..

[39] Brown A., Horobin A., Blount D.G., Hill P.J., English J., Rich A., Pritchard D.I. (2012). Blow fly Lucilia sericata nuclease digests DNA associated with Wound slough/eschar and with Pseudomonas aeruginosa biofilm. Med. Vet. Entomol..

[40] Wang T.Y., Wang W., Li F.F., Chen Y.C., Jiang D., Chen Y.D., Wang A.P. (2020). Maggot excretions/secretions promote diabetic Wound angiogenesis via miR18a/19a—TSP-1 axis. Diabetes Res. Clin. Pr..

[41] Nigam Y., Morgan C. (2016). Does magot Therapy promote Wound healing? The clinical and cellular evidence. JEADV.

[42] Cazander G., Pawiroredjo J.S., Vandenbroucke-Grauls C.M.J.E., Schreurs M.W.J., Jukema G.N. (2010). Synergism between maggot excretions and antibiotics. Wound Repair Regen..

[43] Fijałkowska M., Kowalski M., Koziej M., Antoszewski B. (2021). Elevated serum levels of cathelicidin and β-defensin 2 are associated with basal cell carcinoma. Cent. Eur. J. Immunol..

[44] Bowling F.L., Salgami E.V., Boulton A.J. (2007). Larval therapy: A novel treatment in eliminating methicillin-resistant Staphylococcus aureus from diabetic foot ulcers. Diabet. Care..

[45] Smith F., Dryburgh N., Donaldson J., Mitchell M. (2011). Debridement for surgical wounds. Cochrane Database Syst. Rev..

[46] Wolcott R. (2015). Disrupting the biofilm matrix improves Wound healing outcomes. J. Wound Care.

[47] Szczepanowski Z., Grabarek B.O., Boroń D., Tukiendorf A., Kulik-Parobczy I., Miszczyk L. (2022). Microbiological effects in patients with leg ulcers and diabetic foot treated with Lucilia sericata larvae. Int Wound J..

[48] Nezakati E., Hasani M.H., Zolfaghari P., Rashidan M., Sohrabi M.B. (2020). Effects of Lucilia sericata Maggot Therapy in Chronic Wound Treatment: A Randomized Clinical Trial. Chronic Wound Care Manag. Res..

[49] Eming S.A., Martin P., Tomic-Canic M. (2014). Wound repair and regeneration: Mechanisms, signaling, and translation. Sci. Transl. Med..

[50] Serra R., Rizzuto A., Rossi A., Perri P., Barbetta A., Abdalla K., Caroleo S., Longo C., Amantea B., Sammarco G. (2017). Skin grafting for the treatment of chronic leg ulcers—A systematic review in evidence-based medicine. Int. Wound J..

[51] Burnett L.N., Carr E., Tapp D., Raffin Bouchal S., Horch J.D., Biernaskie J., Gabriel V. (2014). Patient experiences living with split thickness skin grafts. Burns.

[52] Sherman R.A. (2002). Maggot versus conservative debridement therapy for the treatment of pressure ulcers. Wound Repair Regen..

[53] Sherman R.A. (2003). Maggot Therapy for treating diabetic foot ulcers unresponsive to conventional therapy. Diabetes Care.

[54] Gieroń M., Słowik-Rylska M., Kręcisz B. (2018). Effectiveness of maggot debridement therapy in treating chronic wounds–Review of current literature Medical Studies. Stud. Med..

